# Development of the Informed Choice in Mammography Screening Questionnaire (IMQ): factor structure, reliability, and validity

**DOI:** 10.1186/s40359-019-0291-2

**Published:** 2019-03-19

**Authors:** Maren Reder, Eva-Maria Berens, Jacob Spallek, Petra Kolip

**Affiliations:** 1 Bielefeld University, School of Public Health, Department of Prevention and Health Promotion, Universitätsstraße 25, Bielefeld, 33615 Germany; 2 Bielefeld University, School of Public Health, Department of Health Services Research and Nursing Science, Universitätsstraße 25, Bielefeld, 33615 Germany; 3 Brandenburg University of Technology Cottbus-Senftenberg, Department of Public Health, Universitätsplatz 1, Senftenberg, 01968 Germany; 4 University of Hildesheim, Institute of Psychology, Universitätsplatz 1, Hildesheim, 31142 Germany

**Keywords:** Mammography screening, Informed choice, Confirmatory factor analysis, Reliability, Validity

## Abstract

**Background:**

Informed choice is of ethical and practical importance in mammography screening. To assess the level to which decisions regarding such screening are informed is thus imperative, but no specific instrument has been available to measure informed choice in the German mammography screening programme. The aims of this study were to develop the Informed Choice in Mammography Screening Questionnaire (IMQ) and to find first evidence for the factor structure, reliability and validity of its different components.

**Methods:**

The IMQ was sent to 17.349 women aged 50 in Westphalia-Lippe, Germany. The instrument has been developed after consideration of (1) the results of qualitative interviews on decision making in the mammography screening programme, (2) relevant literature on other informed choice instruments and (3) a qualitative study on influencing factors. The IMQ comprises 3 scales (attitude, norms, and barriers), 1 index (knowledge) and singular items covering intention to participate and sociodemographic variables. To assess the psychometric properties of the components of the IMQ, confirmatory factor and item response theory analyses were conducted. Additionally, reliability, validity and item statistics were assessed.

**Results:**

5.847 questionnaires were returned (response rate 33.7%). For attitude, the confirmatory factor analysis supported a one-factor structure. For norms, the model fit was not acceptable. Reliability levels were good with a Cronbach‘s *α* of.793 for attitude (4 items) and.795 for norms (5 items). For barriers, 9 items were deleted because of low discrimination indices; 6 items remained. The hypothesised assumption-subscale and the importance-subscale were confirmed, but these subscales showed poor reliabilities with Cronbach‘s *α*=.525 (4 items) and.583 (2 items). For the knowledge index, item response theory analysis showed that 6 out of 7 items were suitable. Hypotheses concerning the correlations between the different components were confirmed, which supported their convergent and divergent validity.

**Conclusion:**

The results of this study demonstrated that the IMQ is a multidimensional instrument. Further development of the barriers and norms scales is necessary. The IMQ can be utilised to assess the level of informed choices as well as influencing factors.

**Electronic supplementary material:**

The online version of this article (10.1186/s40359-019-0291-2) contains supplementary material, which is available to authorized users.

## Background

Breast cancer is the most common cancer in women in Germany [1]. To reduce mortality from breast cancer and to improve treatment opportunities, a comprehensive mammography screening program for women aged 50 to 69 years was introduced in Germany in 2002 [2]. Fewer women die of breast cancer when they participate in mammography screening, but there is a lot of uncertainty regarding the size of the effect [3]. When offered a screening, in which it is unclear whether the benefits outweigh the harms, it is important that women make informed choices. Especially health services aimed at healthy individuals, which is the case for the mammography screening programme, make informed choices crucial. Being properly informed may reduce the impact of negative consequences. A false-positive screening mammogram may lead to psychological distress lasting for as long as 3 years [4]. It is possible that knowing about the likelihood of false positive screening results could alleviate the stress of a positive result (since one would be aware that the likelihood of cancer is still low). Overdiagnosis is a major harm of mammography screening [5] and can be defined as ‘detecting disease that would not present clinically during the woman’s lifetime’ without participation in mammography screening [6]. Women’s knowledge of possible screening outcomes and their likelihood is a prerequisite for informed choice and of practical and ethical importance [7, 8]. Informed choice serves as quality marker in health care and has become increasingly advocated by many organisations in the last years (e.g. the Institute for Quality and Efficiency in Health Care [9], IQWiG, a German independent scientific institute established under the Health Care Reform 2004). Unfortunately, informed choice is still poorly understood, regarding process as well as outcomes [10].

In spite of the importance of informed choice in mammography screening, there is a notable lack of instruments for measuring this outcome in the mammography screening context. Informed choice comprises the dimensions of relevant knowledge, a decision consistent with personal values and behavioural implementation [11]. Discrepancies between attitude and behaviour may occur due to social pressure and barriers [12]. Therefore, it is important to assess norms and barriers simultaneously with the dimensions of informed choice. Unfortunately, often only knowledge is assessed. The existing knowledge scales vary in their difficulty and their coverage of topics (often lacking critical aspects of mammography screening like overdiagnosis). This leads to uncomparable estimates of women’s knowledge levels.

To assess informed choice in the context of antenatal screening, Marteau et al. [11] developed a groundbreaking instrument. It comprises 8 knowledge items and 4 attitude items and determines uptake via medical records. Michie et al. [12] applied the same instrument successfully in a larger sample, where the knowledge and attitude scale showed to be internally consistent. Based on the assessment in prenatal screening, Mathieu et al. developed two instruments measuring informed choice in mammography screening to evaluate decision aids; one was aimed at women aged 70 [13], one at women aged 40 [14] (i.e., both were developed for women not in the targeted screening age). The instrument for women aged 70 included knowledge, values, and intention [13]. Notably, opposed to Marteau, values were assessed through the values clarity subscale of decisional conflict scale and intention was assessed through a Likert-type format instead of using uptake records as third dimension. In a subsequent trial with 40 year old women, Mathieu et al. [14] assessed knowledge employing a scale adapted from their previous trial [13] for this younger age group and values were assessed with an attitude scale similar to Marteau et al. [11]. A study assessing informed choice in women aged 50 was conducted in the Netherlands [15] relying on expert consultations for the knowledge dimension and on using an attitude scale from a previous prenatal screening informed choice measure [16].

At the time of our study, no specific instrument was available to measure informed choice in the context of the German mammography screening programme. The aim of this study was to develop and psychometrically evaluate an instrument, called Informed Choice in Mammography Screening Questionnaire (IMQ). The IMQ was developed in the context of the study ‘Informed Choice of German and Turkish Women for Participation in the mammography screening programme (InEMa)’ which aimed to assess the level of informed choices in women invited to the mammography screening programme for the first time (see [17]).

## Methods

### Development of the Informed Choice in Mammography Screening Questionnaire

To classify choices as informed, we used the three-dimensional model developed by Marteau et al. [11]. According to this model, an informed choice constitutes a decision based on relevant knowledge, in consistence with individual values and leading to action. However, this model does not incorporate an important predictor of action: the decision/intention. Only using intention as third dimension enables us to assess the informedness of a decision prior to the actual behaviour. This approach of applying the concept of informed choice to intention has been used in previous research (e.g., [11]).

As logic model for the decision process, we chose the reasoned action approach [18]. Based on this model we chose to assess the following constructs in the IMQ: intention, attitude, barriers, and norms. Barriers were assessed as more tangible proxies for control beliefs; advice as proxy for normative beliefs. This was done because the pilot study showed that questions regarding beliefs were not easily understood by the target group and were deemed too abstract. The behaviour of interest was defined according to action, target, context and time [18]: Attending (action) mammography screening (target) as part of the national mammography screening programme to which one was invited (context) in the next three months (time).

The questionnaire was based on qualitative interviews with German and Turkish women, a qualitative study on factors related to mammography screening participation among Turkish women [19], and existing instruments for informed choice and its components. The reasoned action approach [18] provided the basis for our measures of attitude, norms (influence from others), and barriers (perceived barriers). The search for existing instruments on informed choice in mammography screening yielded mostly studies assessing aspects of knowledge. Therefore, also an extensive search was conducted for studies that assessed informed choice in other medical contexts.

Qualitative interviews were conducted to determine what informed choice means for women in the context of (non-)participation in the mammography screening programme and how they arrive at a decision. Four autochthonous and two Turkish women of the mammography screening programme target age group were interviewed. The analysis followed content analysis guidelines [20] and showed that the decision for (non-)participation was usually made only after some time, in which versatile advice, both from physicians and friends, was sought. Participation was also described as the ‘reasonable’ action. It was positively remarked that through the program character, even hard to reach women could be addressed. Being well informed was not a priority, partly due to lack of interest, partly because it was not seen as helpful for the decision.

Study procedures proved feasible in a pretest with 300 invited women. Both comments to the questionnaires and a high proportion of missing responses indicated that questions on objective risk of breast cancer were perceived as problematic.

For the final version, the thematically problematic items were removed from the questionnaire and the questionnaire was considerably shortened. Thus, the final version of the IMQ assessed the three dimensions necessary to form informed choice as well as mapping the decision within a logic model based on the reasoned action approach. The IMQ consists of 3 scales (attitude, norms, and barriers), 1 index (knowledge) and singular items on influencing factors. The German questionnaire was presented as additional file in a previous article [21]. An English translation of the IMQ components is provided in Additional file 1.

### Measures

*Informed choice* was assessed through the following dimensions according to the 3-dimensional classification model of Marteau et al. [11]: knowledge (sufficient/insufficient), attitude (positive/negative) and intention (yes/no). An informed decision is present, if a woman on the basis of sufficient knowledge either intends participation while having a positive attitude or rejects participation in the screening program while having a negative attitude.

*Intention to participate in the mammography screening programme* was measured with two items: (1) intention to participate in a screening mammography within the next 3 months (yes/no/undecided), and (2) type of screening (opportunistic screening/mammography screening programme). These items reflect the German context in which the mammography screening programme runs parallel to opportunistic screening. Three months was defined as time frame for participation because our questionnaire was timed to arrive once the women had received the invitation to the mammography screening programme (which usually suggests an appointment within the next 3 months). For the calculation of informed choice, intention was dichotomised as ‘participation in the mammography screening programme’ and ‘no participation in any mammography for early detection’. All other intentions (opportunistic screening) were excluded from the calculation. 5.3% of our sample decided to have opportunistic mammography screening [21]. We excluded these women because in this age group in Germany, women having a mammogram outside of the screening programme will either have a high risk profile or a suspected breast cancer (although both concepts may be somewhat extendible undermining the idea that opportunistic screening in a normal risk population should not exist parallel to the programme).

*Attitude* was measured using four items developed by Marteau [11] in the context of antenatal screening and according to the reasoned action approach of Fishbein and Ajzen [18]. Three semantic differentials (important/unimportant; a good thing/a bad thing; beneficial/harmful) assessed instrumental attitude (i.e., consequences). One semantic differential assessed experiential (i.e, the anticipated experience) attitude (comfortable/uncomfortable). Women were asked to rate the statement ‘To participate in the mammography screening programme is...’ on the above described four semantic differentials (discrete visual analog scale from -2 to +2).

*Knowledge* was assessed with an index comprising 7 multiple choice items based on the knowledge questions of Mathieu et al. [14]. The questions covered the following: (1) screening for people without symptoms; (2) frequency of positive screening results; (3) false positives; (4) false negatives; (5) diagnoses with the mammography screening programme; (6) breast cancer deaths without the mammography screening programme; and (7) overdiagnosis and overtreatment. The items had two to four answer options of which one was correct. Missing responses and ‘Don’t know’ responses were categorised as incorrect.

*Barriers* were measured using 15 items rated on a five-point discrete visual analog scale with the anchors of ‘agree’ and ‘disagree’. Based on the questions regarding barriers in other studies [22–25], we identified two topic areas: (1) assumptions about the mammography screening and (2) importance of the mammography screening. The scale we constructed comprised these two subscales. The items (see Table 1) B1 and B7 stem from Lee et al. [22], B2 and B10 from Champion et al. [23], B5, B12 and B15 from Tyndel et al. [24], and B11, B13 and B14 from Strong et al. [25]. B8 and B9 were developed specific to the context of the German mammography screening programme, and finally B3, B4 and B6 stem from our interview data. Items 8 and 9 were reverse coded so that for each item a higher number indicated a stronger barrier. The individual items took the values 0 (no barrier) to 4 (strong barrier).

**Table 1:** Item analysis of all attitude, barriers, and norms items

Scale	Item no.	Item	*n*	Difficulty index	Variance	Discrimination index	Factor loading	Cronbach’s *α*
Attitude	A1	Important - Unimportant	5234	87.7	0.64	.722	.908	.793
	A2	A good thing - A bad thing	5156	89.1	0.51	.758	.930	
	A3	Comfortable - Uncomfortable	5000	47.5	1.14	.374	.375	
	A4	Advantageous - Disadvantageous	5075	82.6	0.69	.678	.752	
Barriers: assumptions about MSP	B1	Uncomfortable with body being touched during examination	5244	34.1	1.85	.301	.569	.525 (only items B1, B2, B3, B6)
	B2	Afraid of pain during MS	5253	35.9	2.09	.367	.666	
	B3	Got conflicting advice regarding MSP	5194	25.5	1.95	.349	.345	
	B4	See the course of disease as predetermined	5171	22.3	1.51	.229	-	
	B5	Don’t want to know whether something is wrong	5238	9.3	0.85	.255	-	
	B6	Insecure what to expect	5229	35.4	2.15	.288	.471	
	B7	Radiation of MS is harmful	5146	34.3	1.42	.299	-	
	B8	Feel obliged through the invitation	5237	74.6	2.15	-.210	-	
	B9	Have trust in the MSP	5235	31.1	1.79	.115	-	
Barriers: importance of MSP	B10	Other problems more important than MS	5249	15.9	1.20	.326	.730	.583 (only items B10, B11)
	B11	No time for appointment	5219	7.4	0.65	.421	.517	
	B12	Am on holiday/abroad	5163	1.3	0.14	.205	-	
	B13	Have language problems	5188	1.3	0.15	.193	-	
	B14	Financial costs are too high	5097	5.8	0.56	.345	-	
	B15	Problems getting to the screening unit	5198	4.0	0.41	.332	-	
Norms	N1	Gynecologist	2992	94.1	0.45	.539	.519	.795
	N2	General practitioner	1130	92.2	0.54	.560	.572	
	N3	Partner	2306	91.7	0.60	.590	.673	
	N4	Relatives	1965	83.7	1.03	.637	.732	
	N5	Friends/acquaintances	2812	81.3	1.12	.621	.610	

*Note.* MSP: mammography screening programme. MS: mammography screening

*Norms* were assessed with five items rated on a five-point discrete visual analog scale, ranging from ‘advise’ to ‘disadvise’ with the additional option of ‘no advice’. These items assessed the direction of advice of doctors, family, and friends. The individual items took the values -2 (disadvice) to +2 (advice). Items with the answer option ‘no advice’ were treated as missing values for the scale calculation.

*Singular items* Decision confidence and self-rated knowledge were each assessed with a 5-point discrete visual analog scale item. Mammography uptake was assessed at 3-months follow-up with one item with the response options (1) participation in the mammography screening programme in the last 3 months, (2) opportunistic screening, and (3) no screening mammography.

### Study design and data collection procedures

The German version of the IMQ, a 12-page, self-administered paper-and-pencil questionnaire, was sent to 17.349 women (1.789 of which additionally received a Turkish questionnaire) aged 50 in Westphalia-Lippe, a region in the Federal State of North-Rhine Westphalia, Germany, from October 2013 to July 2014. The IMQ was mailed to the women 1 to 2 months after their 50th birthday when they were expected to receive their invitation to the mammography screening programme by the regional mammography organisation and thus have to make a choice for or against mammography screening programme participation. Participants were informed about the purpose of the study, the voluntary and anonymous nature of the data collection, and the analysis procedure. Written informed consent was obtained. The study was cleared by the ethical committee of the Medical Faculty of Muenster University (2012-268-f-S). The data collection is described in more detail in the study protocol [17].

### Statistical analysis

The data were entered manually in Microsoft Access and imported into SPSS version 22.0 (IBM Corp., Armonk, NY) and Mplus version 7.31 (Muthén & Muthén, Los Angeles, CA) for analysis. To assess the psychometric properties of the components of the IMQ, three steps of analysis were conducted: (1) item statistics, (2) confirmatory factor / item response theory analyses, and (3) correlations to assess validity.

**Discrete visual analog scale items** For the scales with 5-point discrete visual analog scale items (attitude, norms, and barriers), the item-discrimination index and the item-difficulty index were calculated in SPSS. The item-difficulty index indicates how many women responded to an item in a positive/agreeing direction. Medium item difficulty indices enlarge the probability for high variance and thus maximum differentiation [26] while a wide variation in difficulty indices allows differentiation across the whole spectrum of the construct.

The item-discrimination index indicates how adequately an item discriminates between high and low scorers and is calculated as corrected item scale correlation. Discrimination indices of <.30 were considered low, of.30−.50 medium, and of >.50 high. For item selection, the items should at least have an item discrimination index of >.30 [26]. Items with negative item discrimination-index are unsuitable for scale construction [26].

We conducted maximum likelihood confirmatory factor analyses to test the hypothesised factorial structure of the scales. Model fit was assessed using the following model fit indices and cut-off values: Comparative Fit Index (CFI) ≥.90, Tucker-Lewis Index (TLI) ≥.90, Root Mean Squared Error of Approximation (RMSEA) ≤.08 [27], and Standardised Root Mean Squared Residual (SRMR) <.09 [28].

Given our large sample size, it was likely that the *χ*^2^-tests would become significant [27]. Therefore, the other indices were used to assess the quality of model fit. Completely standardised factor loadings were reported. Loadings of >.71 were considered excellent, >.63 very good, and >.55 good [29].

To assess reliability, we calculated Cronbach’s *α*. Levels >.70 indicated acceptable reliability [30].

**Dichotomously scored multiple choice items** For the knowledge index consisting of 7 multiple choice items (with responses either scored as right or wrong), we modelled one- and two-parameter logistic models in M-Plus and compared these to establish whether item discrimination is equal between items. We used the Mean- and Variance-adjusted Weighted Least Square estimator to obtain absolute model fit indices.

Two-parameter logistic item response theory models reduce response patterns to a latent trait score (theta) and provide information about item discrimination and item difficulty. Thus, they describe the relationship between a latent construct, which the scale is supposed to measure, the properties of the items constituting this scale, and responses to the individual items [31]. Our model thus assumes the items posses different abilities to discriminate women with high levels of the underlying construct knowledge from women with low levels. We assessed scale dimensionality through the above described model fit indices to determine acceptability of the model fit.

In two-parameter logistic models, items with higher discrimination count more towards the underlying construct (*θ* = knowledge) reflecting the strength of association of an item with its construct. This means that item discrimination indicates how well an item separates women with knowledge below the item location from women with knowledge above the item location. The steeper the slope of the item characteristic curve in its middle section (i.e., where the probability of a correct answer is.5), the higher the discrimination [31]. Conversely, for items with low discrimination this means that a small change in the underlying construct knowledge, leads only to a small change in the probability of answering this item correctly. Item difficulty indicates where the item functions along the underlying construct knowledge (i.e., knowledge level [location on the latent trait] at which a woman has a.5 probability of answering the item correctly).

**Validity** The convergent and divergent validity of the components represented in the IMQ were investigated by calculating their intercorrelations. Two-sided significance was determined. Correlations of.1 to <.3 were regarded as small, correlations from.3 to.5 as moderate, and correlations of >.5 as large. Correlations <.1 were considered negligible and not interpreted. We hypothesised small correlations between attitude, barriers and norms. Knowledge was assumed to correlate with no other outcome.

According to a recent meta analysis [32], moderate correlations exist between intention and behaviour, instrumental attitude and intention, and injunctive norms and intention. Autonomy and intention showed a small correlation [32]. Therefore, we assumed intention to have small to moderate correlations with attitude, barriers, and norms. The knowledge index was hypothesised to correlate with self-rated knowledge. Informed choice was assumed to be associated with decision confidence. Additionally, to assess the predictive validity of the components, we assessed their correlations with mammography uptake.

## Results

### Participants

5847 women (33.7%) responded to the questionnaire. Women who had ever had breast cancer (*n*=183), who had already participated in the MSP (*n*=256), and who had completed the Turkish questionnaire (*n*=114) were excluded, since this publication reports the psychometric properties of the German version of the IMQ. So overall, 5293 German questionnaires were used to calculate the psychometric properties of the IMQ. Of those, 36.4% had a university or university of applied sciences entrance qualification (equalling 11 to 13 years of education). A further 41.3% had received an intermediate school certificate (equalling 10 years of education). 19.0% had obtained a secondary general school certificate (equalling 9 years of education). 1.2% had left school without certificate. 91.7% had no migration background, 4.2% were resettlers, and 1.0% had a Turkish migration background.

### Item indices

In Table 1, the item difficulty, variance, and item discrimination index are shown. Regarding item difficulty, the attitude scale had favourable values although A1, A2 and A4 were very similar. The barriers scale had some very low item difficulties. The variance of B12 and B13 was very low, as hardly any women regarded these items as barriers. The barriers scale had many items with an item discrimination index below the cut-off level of.30. The item discrimination index of B8 was the only negative index rendering this item inadequate for scale construction. The items of the norms and attitude scale had all acceptable indices, although the experiential attitude item (A3) was just above the cut-off.

### Factorial structure

For attitude, the factor loadings ranged from.375 to.930 (see Table 1). Overall, three factor loadings were classified as excellent. The factor loading of A3 did not reach the criterion of a good factor loading. This can be explained by the fact that it was the only item assessing experiential attitude. The *χ*^2^-test of model fit was significant (*χ*^2^=78.920,*df*=2, *p*<.001). The CFI (*CFI*=0.992), the TLI (*TLI*=0.976), and the SRMR (*SRMR*=0.021) were better than the cut-off values. The RMSEA (*RMSEA*=0.090, 90%- *CI*=0.074 to 0.108) was below the thresholds for acceptable fit. Overall, the factor structure of the scale was considered acceptable.

For barriers, all items with an item discrimination index of <.30 and a variance of <0.50 were excluded. Item 6 was kept on substantive considerations. The remaining items (B1, B2, B3, B6, B10, B11) were assessed in a confirmatory factor analysis comprising the two subscales assumptions and importance. The factor loadings ranged from.345 to.730 (see Table 1). Overall, one factor loading was classified as excellent, one as very good, and one as good while three factor loadings did not reach this criterion. The *χ*^2^-test of model fit was significant (*χ*^2^= 74.835, *df*=8, *p*<.001). The CFI (*CFI*=0.976), the TLI (*TLI*=0.954), the RMSEA (*RMSEA*=0.042, 90%- *CI*=0.034 to 0.051), and the SRMR (*SRMR*=0.023) indicated an acceptable fit.

For norms, the factor loadings ranged from.519 to.732 (see Table 1). Overall, one factor loading was classified as excellent, one as very good, and two as good while one factor loading was just below this criterion. The *χ*^2^-test of model fit was significant (*χ*^2^= 273.54, *df*=5, *p*<.001). The CFI (*CFI*=0.846), the TLI (*TLI*=0.692), and the RMSEA (*RMSEA*=0.117, 90%- *CI*= 0.105 to 0.129) were below the thresholds for acceptable fit. Only the SRMR (*SRMR*=0.082) indicated an acceptable fit. Overall, the factor structure of the scale was not considered acceptable.

### Reliability

For attitude (4 items), the internal consistency was good with.793 (Cronbach’s *α*), especially considering its short scale length (see Table 1). For barriers, the internal consistency was poor. The assumptions-subscale (4 items) had an internal consistency of.583, the importance-subscale (2 items) of.525. For norms (5 items), the reliability was good with.795.

### IRT results

**Fig. 1:**
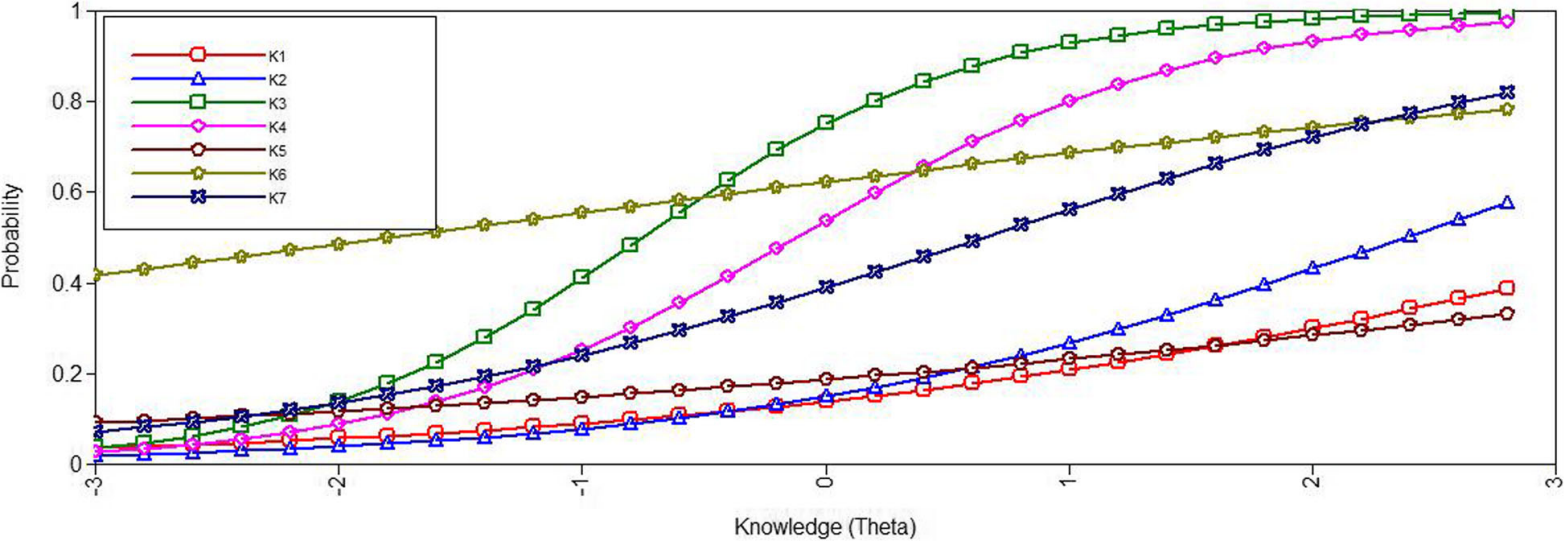
Item characteristic curves of the knowledge items (2-parameter-logistic-model)

For the knowledge index, we tested the unidimensionality assumption of IRT by fitting a 1-factor 2-parameter-logistic IRT model. The Fit-information indicated an inadequate model fit. The *χ*^2^-test of model fit was significant (*χ*^2^= 362.80, *df*=14, *p*<.001), the CFI (*CFI*=0.729) and the TLI (*TLI*=0.594) were below the thresholds for acceptable fit. Only the RMSEA (*RMSEA*=0.069, 90%- *CI*= 0.063 to 0.075) indicated an acceptable fit. The corresponding item characteristic curves that represent the respondents’ knowledge (latent factor) in relation to the probability of answering an item correctly are depicted in Fig. 1.

After evaluating the discrimination and difficulty parameters of the individual items (see Table 2), we excluded item 6 (likelihood to die of breast cancer larger, smaller, or equal for women participating in the mammography screening programme). However, consideration should be given to retaining the question as a stand-alone item, since it addresses an important knowledge aspect. The resulting model (*χ*^2^= 94.20, *df*=9,*p*<.001; *CFI*=0.915; *TLI*=0.858; *RMSEA*=0.042, 90%- *CI*= 0.035 to 0.050) indicated an acceptable fit. The remaining six items can thus, be assumed to have sufficient unidimensionality.

**Table 2:** Item difficulty and discrimination for the knowledge index

Item	Item	7 item model		6 item model*	
No.		Item discrimination (SE)	Item difficulty (SE)	Item discrimination (SE)	Item difficulty (SE)
K1	When to participate in the MSP (*No breast complaints*/ Breast complaints/ In both cases)	0.267 (0.036)	4.040 (0.520)	0.256 (0.036)	4.202 (0.560)
K2	Number of women receiving a positive result (1-20 of 200/ 21-50 of 200/ *51-100 of 200*/ 101-200 of 200)	0.428 (0.038)	2.411 (0.192)	0.403 (0.037)	2.537 (0.212)
K3	Positive MSP result means breast cancer (Yes/ *No*)	0.810 (0.060)	-0.782 (0.046)	0.805 (0.061)	-0.785 (0.047)
K4	MSP discovers every breast cancer (Yes/ *No*)	0.710 (0.049)	-0.126 (0.030)	0.812 (0.062)	-0.115 (0.028)
K5	More likely to get the diagnosis breast cancer (*Women participating in the MSP*/ Women not participating in the MSP/ Both the same)	0.202 (0.031)	4.442 (0.671)	0.120 (0.030)	7.341 (1.836)
K6	More likely to die of breast cancer (Women participating in the MSP/ *Women not participating in the MSP*/ Both the same)	0.208 (0.028)	-1.505 (0.209)	-	-
K7	Existence of overtreatment (*Yes*/ No)	0.450 (0.033)	0.623 (0.057)	0.433 (0.033)	0.644 (0.060)

*Note.* Correct answers in italics. * Item 6 was excluded

The items of the 6-item knowledge index covered a fair spectrum of item difficulty. Items with the highest discrimination capacity were K3 and K4. K1 and K5 showed very low item discrimination. Table 2 summarises the discrimination parameters and difficulty parameters.

In a second step, we specified a 1-parameter-logistic model (*χ*^2^= 324.490, *df*=14,*p*<.001; *CFI*=0.690; *TLI*=0.668; *RMSEA*=0.065, 90%- *CI*= 0.059 to 0.071). This model fitted worse than the 2-parameter-logistic model as the difference test showed (*χ*^2^= 200.729, *df*=5,*p*<.001). This indicates that item discrimination is not equal across items.

### Validity

Validity was assessed through correlations between the components of the IMQ and uptake (Table 3). Kendall’s *τ* was chosen as correlation coefficient, as no variable met the assumption of normal distribution. No intercorrelation exceeded.85, suggesting divergent validity of all components.

**Table 3:** Intercorrelations between the scales attitude, barriers (assumptions and importance), norms, the knowledge index, intention, and uptake

Scale/Index	*n*	Barriers: assumptions about MSP	Barriers: importance of MSP	Norms	Knowledge	Intention	Uptake
Attitude	5274	**-.287** (*p*<.001)	**-.250** (*p*<.001)	**.273** (*p*<.001)	**-.085** (*p*<.001)	**.284** (*p*<.001)	**.300** (*p*<.001)
Barriers: assumptions about MSP	5139	-	**.224** (*p*<.001)	**-.112** (*p*<.001)	**-.024** (*p*=.018)	**-.032** (*p*=.027)	**-.047** (*p*=.003)
Barriers: importance of MSP	5262	-	-	**-.157** (*p*<.001)	**.032** (*p*=.003)	**-.178** (*p*<.001)	**-.171** (*p*<.001)
Norms	4169	-	-	-	**-.051** (*p*<.001)	**.166** (*p*<.001)	**.175** (*p*<.001)
Knowledge	5293	-	-	-	-	**-.039** (*p*=.007)	**-.044** (*p*=.005)
Intention	4893	-	-	-	-	-	**.539** (*p*<.001)
Uptake	4165	-	-	-	-	-	-

*Note.* Kendall’s *τ* correlation coefficients for all scales and the knowledge index. Point-biserial correlation coefficients for intention and uptake. *p*-values are for two-tailed significance. Significant correlations are bolded

Attitude had a weak negative correlation with both barrier subscales and a weak positive correlation with norms. The barrier subscales had weak negative correlations with norms. The mediators of the logic model, thus, correlated weakly with each other supporting the difference between the constructs and being consistent with the logic model – it has to be noted though that barriers had shown a 2-factor structure in a CFA and that the two barrier subscales only showed a weak correlation among themselves.

The weighted knowledge index, which resulted from the previously conducted item response theory analysis showing that a 2-parameter-logistic model had a better model fit, correlated negligibly with attitude, norms, and the assumptions- and importance-subscales. Intention showed a negligible correlation with knowledge and only weak positive correlations with norms and attitude. Intention and the importance-subscale showed a weak negative correlation. This indicates that intention is most strongly influenced by attitude but also that none of the constructs serves well as a singular predictor of intention. This supports the conceptualisation of informed choice as multidimensional classification model.

Intention at T1 and uptake at T2 correlated strongly with.539 (*p*<.001). Attitude showed a moderate, knowledge a negligible correlation with uptake. The importance-subscale showed a weak negative correlation, norms a weak positive correlation.

Decision certainty and informed choice correlated with.049 (*p*<.001) indicating a negligible association. Self-rated knowledge and knowledge correlated with.181 (*p*<.001) indicating only a weak association.

Additionally, we conducted a logistic regression, *R*^2^ =.116 (Nagelkerke), *χ*^2^(8) = 64.830, *p*<.001, to assess how well the different components predicted intention. All predictors were significant (assumptions-subscale: *B*=.068,*p*<.001, OR=1.071 [95% C.I. 1.046 to 1.096]; importance-subscale: *B*=−.123,*p*<.001, OR= 0.884 [95% C.I. 0.841 to 0.930]; norms: *B*=.060,*p*<.001, OR=1.062 [95% C.I. 1.032 to 1.093]; attitude: *B*=.203,*p*<.001, OR=1.225 [95% C.I. 1.185 to 1.267]). Attitude showed the highest odds ratio; only the importance-subscale showed a negative effect on intention.

## Discussion

In this study, a measure of informed choice was developed and its psychometric properties were determined. To evaluate the psychometric properties of the IMQ components, the sample size was sufficiently large. Attitude and barriers possessed an acceptable factor structure. However, this did not apply to norms. Attitude and norms showed an acceptable internal consistency. The barriers subscales reached only low internal consistency values. The knowledge index showed sufficient unidimensionality after excluding item 6. For some items, item discrimination was low, but overall the 6-item index of knowledge showed acceptable item parameters. The evaluation of the correlation pattern supported the validity assumptions of the logic model as well as those of the classification model of informed choice.

Overall, it has to be noted that the norms and barriers scales are not essential to an instrument assessing informed choice, as they are not part of this classificatory model. Nevertheless, these scales are important within the logic model and can be of value in both practical and research contexts (e.g., to be better able to predict intention).

The *knowledge index* comprised only some pieces of information about the mammography screening programme. These were carefully selected, but did not cover the entire spectrum of decision-relevant facts. The unfamiliar questionnaire format may have been difficult for some women leading to underestimation of their knowledge level. Knowledge instruments are hardly comparable across studies, since different aspects, difficulties and answer formats are used. Another insecurity in determining sufficient knowledge is that there are no clear guidelines as to what level constitutes sufficient knowledge [12]. Since for the calculation of informed choice, dichotomisation is vital, we use the mid-point in congruence with other studies [12, 15], while it has to be noted that other researchers have proposed the median [11].

In concordance with van Agt [15] considering psychometric methods - adapted from the field of educational tests - beneficial for the development of knowledge instruments, we used item response theory analyses for our knowledge index. Similar to our results, Michie et al. [33] used item response theory analysis for their knowledge items, which showed that the items (with one exception) reflected a spread of difficulty and discriminate between women. The 2-parameter-logistic model fits our data better than the 1-parameter-logistic model. This implies that it is important which items are answered correctly rather than counting only the number of correct items, as had been our initial intention for this index. Therefore, a summary index cannot be recommended as method but instead either (1) a latent approach (which would allow 2-parameter-logistic modelling) or (2) a summary index of the weighted items (i.e., weighted by the discrimination parameters [34]). Unfortunately, to our knowledge no latent approach for the calculation of informed choice has been proposed to date. A summary index of the weighted items would still allow dichotomisation at the weighted indexes midpoint (weighted scale range 0 to 2.8; midpoint 1.4) for the calculation of informed choice.

The four semantic differentials assessing *attitude* cover only few - albeit important - advantages and disadvantages of the mammography screening programme. In addition, the different attitude aspects (instrumental and experiential) may be weighted differently by each woman, which was not assessed in our questionnaire. The items indicated a good internal consistency of.79. Similar items in other research reached a Cronbach’s alpha between.77 and.85 [11, 12, 15, 33]. For assessment of informed choice, the continuous construct of attitude has to be dichotomised: A score of ≥0 is to be classified as positive attitude. Similar to van Agt, where, with a scale range of 0 to 24, >12 was categorised as positive attitude [15].

Regarding *barriers*, we confirmed our hypothesized two factor solution. Similar to our results, Kwok et al. found in the factor analysis of their 7-item barrier scale that it comprised two factors: psychological and practical barriers [35]. This matches our two subscales contentwise: our ‘assumptions about the mammography screening programme’ subscale shows similarity to Kwok et al.’s psychological subscale while our ‘importance of the mammography screening programme’ subscale mirrors what Kwok et al. termed practical barriers.

*Norms* did not have a reasonable model fit. This may be a result of the high proportion of no advice responses or reflect an inhomogeneity of the different important others whose advice may be sought.

A limitation for determining informed choice was that *intention* is not equivalent to behavioural implementation (we found a correlation of.539). This is represented in the logic model but not the classification model of informed choice. Theoretically, both intention or behaviour may serve to calculate informed choice [11]. Nevertheless, this necessarily entails a proportion of women not acting as intended, i.e. they cannot be assigned to one category. Nevertheless, intention can be seen as an appropriate construct to calculate informed choice because the behavioural implementation may be influenced by organisational factors, which occur only after the decision was made.

The *associations* between the components of the IMQ, were comparable to previous research. Attitude was associated with intention similar to previous research on the association of attitude and uptake [33, 35]. The norms items showed little variance. Most people giving advice advised the women to have mammography screening. This may be one reason for the weak correlation of norms and intention. Attitude and knowledge have previously been reported to be not associated [33]. Knowledge did not predict uptake [33] which is similar to our finding of a negative negligible association between knowledge and intention. Informed choice had a negligible correlation with decision certainty. In other research, Michie et al. [12] were able to demonstrate that women, whose decision for participation in Down syndrome screening was informed, felt better informed and supported six weeks after the screening than women whose decision was uninformed. Their research thus supports the validity of a similarly calculated informed choice although comparison across screening types may not be warranted. Future research is needed regarding the predictive validity of our IMQ on decision regret and satisfaction with screening outcomes.

A *general limitation* of this study was that the questionnaire has been applied to a very homogeneous population: Only women aged 50 in Westphalia-Lippe, who had already received an invitation to the MSP and did not have a history of breast cancer were included in the analyses. It may not be appropriate to use the IMQ for women who are not immediately facing a mammography screening decision or are not first-time-invitees. Women who intend to participate in mammography screening may have been more likely to participate in the study as they were interested in the subject and willing to confront themselves with this sensitive topic. Accordingly, results may not be representative of the general population of women invited. Our sample had a higher education level than the population of women aged 50 to 54 in North Rhine-Westphalia. 36.4% of the women in our study had a university or university of applied sciences entrance qualification compared to 32.9% in the population [36]. 43% had an intermediate school certificate in our sample while only 32.5% of the population have this educational degree [36]. Contrastingly, only 19.0% of our sample had obtained a secondary general school certificate compared to 27.8% in the population [36]. The percentage of women without migration background was higher than in women aged 50 to 54 in North Rhine-Westphalia (91.7% in our sample compared to 80.1% [37]). The percentage of resettlers was lower in our sample (4.2% compared to 9.7% of women of similar age in North Rhine-Westphalia [37]). As could be expected, since we did only include the German questionnaires in our analysis, the percentage of women with a Turkish migration background was lower than in the population (1.0% compared to 2.3% [37]). The response rate of 33.7% was similar to other studies on mammography screening in Germany [38, 39]. Future research should evaluate the IMQ in a more diverse group of women. Finally, the cross-sectional study design limited our ability to capture the dynamics of the decision making process although our questionnaire was timed to arrive at the time of decision making. We know from the qualitative interviews we conducted for questionnaire development that the time of decision making can vary widely and that sometimes the women do not experience mammography screening programme participation as a decision they have to make but rather as a matter of course.

## Conclusion

The present study made a contribution in the area of informed choice by developing the IMQ and evaluating its psychometric properties. The IMQ has the potential to become an important tool for researchers and healthcare providers who are working with women trying to decide whether participation in the mammography screening programme is the right choice for them. The IMQ can identify women who made an uninformed choice so they can receive more decisional support or support buffering the negative effects of uninformed choices. The questionnaire can also be used to evaluate interventions targeting informed choice or its components. An important goal of our research is to raise informed choice to the level of a standard outcome to be included in studies on participation in mammography screening. Having developed an adequate questionnaire, is an important step in this direction.

## Additional file

Additional file 1 English version of the Informed Choice in Mammography Screening Questionnaire (IMQ). (PDF 42 kb)

## Abbreviations

CFI: Comparative fit index
IMQ: Informed choice in mammography screening questionnaire
RMSEA: Root mean squared error of approximation
SRMR: Standardised root mean squared residual
TLI: Tucker-Lewis index
