# Supplementary material for: Development of the Informed Choice in Mammography Screening Questionnaire (IMQ): factor structure, reliability, and validity
Source: BMC Psychol. 2019 Mar 19;7:17. doi: 10.1186/s40359-019-0291-2 (PMC6423759; doi:10.1186/s40359-019-0291-2)
Supplement: Supplementary file 1 — English version of the Informed Choice in Mammography Screening Questionnaire (IMQ). (PDF 42 kb) [file 40359_2019_291_MOESM1_ESM.pdf]

Informed Choice in Mammography Screening Questionnaire (IMQ)

Please note that this a translation of the German version of the IMQ. No back translation of the English version of the questionnaire has been conducted. All results described in the Article pertain to the German version of the IMQ.

Attitude

To participate in the Mammography Screening Programme is...
A1) Important O O O O O Unimportant
A2) A good thing O O O O O A bad thing
A3) Comfortable O O O O O Uncomfortable
A4) Advantageous O O O O O Disadvantageous

Barriers: assumptions about MSP

Do you agree with the following statements?

B1) I feel uncomfortable with my body being touched during the examination.
Agree O O O O O Disagree

B2) I am afraid of experiencing pain during the mammography screening.
Agree O O O O O Disagree

B3) I received conflicting advice regarding the Mammography Screening Programme.
Agree O O O O O Disagree

B4) I see the course of disease as predetermined.
Agree O O O O O Disagree

B5) I would rather not know whether something is wrong.
Agree O O O O O Disagree

B6) I am unsure what to expect.
Agree O O O O O Disagree

B7) The radiation of mammography screening is harmful.
Agree O O O O O Disagree

B8) I feel obliged through the invitation.
Agree O O O O O Disagree

B9) I have trust in the Mammography Screening Programme.
Agree O O O O O Disagree

Barriers: importance of MSP

Do you agree with the following statements?

B10) I have other problems that are more important than a mammography.
Agree O O O O O Disagree

B11) I have no time for the appointment.

Agree O O O O O Disagree

B12) I am on holiday/abroad.

Agree O O O O O Disagree

B13) I have language problems.

Agree O O O O O Disagree

B14) The financial costs are too high for me.

Agree O O O O O Disagree

B15) I have problems getting to the screening unit.

Agree O O O O O Disagree

Norms

Did the following persons give you advice in favour or against participation in the Mammography Screening Programme?

N1) My gynaecologist

Advise O O O O O Disadvise O No advice

N2) My general practitioner

Advise O O O O O Disadvise O No advice

N3) My partner

Advise O O O O O Disadvise O No advice

N4) My relatives

Advise O O O O O Disadvise O No advice

N5) My friends/acquaintances

Advise O O O O O Disadvise O No advice

Knowledge

K1) When does one participate in mammography screening?

- If one is healthy
- If one recognises a change or knot in the breast
- In both cases
- Don't know

K2) Please imagine the following: 200 women participate in the Mammography Screening Programme for 20 years. How many women will receive a positive result through the screening programme that requires further diagnostics?

- 1-20 of 200
- 21-50 of 200
- 51-100 of 200
- 101-200 of 200
- Don't know

K3) Does a positive mammography screening result mean that a woman has breast cancer?

- Yes
- No
- Don't know

K4) Does mammography screening discover every breast cancer?

- Yes
- No
- Don't know

K5) Who is more likely to get the diagnosis breast cancer?

- Women participating in the Mammography Screening Programme
- Women not participating in the Mammography Screening Programme
- Both the same
- Don't know

K6) Who is more likely to die of breast cancer?

- Women participating in the Mammography Screening Programme
- Women not participating in the Mammography Screening Programme
- Both the same
- Don't know

K7) Are there women, who have been treated for breast cancer, even though the breast cancer would never have caused problems during their lifetime?

- Yes
- No
- Don't know

Intention

I1) I intent to participate in screening mammography within the next 3 months.

- Yes
- No

I2) Where will you have the mammography screening?

- At a medical practitioner (gynaecologist, radiologist, GP)
- Following an invitation to the Mammography Screening Programme

Uptake

In the past 3 months, I have…

- participated in the Mammography Screening Programme to which I have been invited
- received a screening mammography through a medical practitioner (gynaecologist, radiologist, GP)
- had no screening mammography
